# Stigma predicts health-related quality of life impairment, psychological distress, and somatic symptoms in acne sufferers

**DOI:** 10.1371/journal.pone.0205009

**Published:** 2018-09-28

**Authors:** Jamie Davern, Aisling T. O’Donnell

**Affiliations:** Department of Psychology and Centre for Social Issues Research, Faculty of Education and Health Sciences, University of Limerick, Limerick, Republic of Ireland; San Gallicano Dermatologic Institute, ITALY

## Abstract

Acne vulgaris has been associated with deficits in psychological well-being and health-related quality of life. Few studies have investigated how stigma contributes to our understanding of the well-being of acne sufferers, although it is clear that acne is stigmatized and stigmatization is associated with impaired well-being. The current study aimed to investigate the ability of perceived stigma to predict health-related quality of life, psychological distress, and somatic symptoms over and above established predictors. University students and staff suffering from acne completed self-report measures online. Hierarchical multiple regression analyses showed that perceived stigma significantly contributed to the prediction of all three well-being measures, over and above the effects of gender, acne severity, acne location, and use of medication. Indeed, perceived stigma made the largest unique contribution to predicting well-being. Our findings suggest that interventions that attempt to counter stigma could also improve the overall well-being of people affected by acne.

## Introduction

The skin is a highly prominent feature of the human body, is an important element of perceived attractiveness, and is often used to denote information about a person’s health, age and background. Acne vulgaris is a skin condition that affects up to 80% of the population during adolescence [1] and is characterized by the manifestation of whiteheads, blackheads, pustules, papules and cysts that vary in frequency and severity depending on the extent of the sufferer’s condition. Although adolescents are most commonly afflicted by acne, the condition has been reported to affect 10.8% of children between the ages of 5–13 years and 12.7% of adults aged over 59 [2]. The condition has also been associated with several consequences for more general well-being, including depression [3–5], decreased self-confidence [6], fatigue [7], poorer body image satisfaction [8, 9], and increased suicidal ideation [10]. The aspects of the acne experience that contribute most to predicting such impaired well-being have not yet been adequately explored. In the current paper, we investigate the ability of a social psychological factor, stigma, to help predict relevant measures of impaired well-being in those who suffer from acne.

In the literature on dermatological conditions, including acne, health-related quality of life is a well-being measure that has received substantial attention. It has been defined as the ‘functional effect of an illness and its consequent therapy upon a patient, as perceived by the patient’ [11]. To expand further, health-related quality of life is a multidimensional construct that accounts for the impact of an individual’s condition on psychological, physical and social domains of well-being [12]. Measuring dermatology-specific health-related quality of life allows researchers to assess the efficacy of treatment options and compare the impact of acne to other skin conditions [13]–and understanding the factors that predict health-related quality of life is important in this. Evidence from existing research suggests that acne has wide-ranging effects, including a negative association with health-related quality of life [14–15].

In seeking to understand this effect, to date, researchers have identified a diverse range of factors that predict the health-related quality of life of acne sufferers; these factors are mainly demographic and practical in nature. For instance, gender differences have been found in past research, with females tending to experience greater impairment than males [15–17]. In some studies, individuals with greater acne severity have been found to have decreased health-related quality of life compared to those with milder acne [1, 18], though other studies have documented that the effects of severity are not important [4, 19]. Additionally, it has been suggested that patients’ perceptions of acne severity are more important to consider than physician-reported severity, due to the consequences that acne has on body image and self-confidence [20]. Acne location also appears to be important: acne mostly affects the face, and individuals with facial acne—in comparison to those with truncal acne—have been shown to have poorer body image satisfaction [9], higher feelings of unattractiveness [21] and decreased health-related quality of life [22]. Finally, successful response to treatments such as isotretinoin [16] and erythromycin-zinc [17] has been demonstrated to have a positive impact on health-related quality of life.

As such, the predictive utility of these demographical and practical factors in determining levels of health-related quality of life in acne sufferers is clear, but there has been less focus on factors that could be considered social psychological or social structural. We argue that one aspect of acne sufferers’ experiences that has not received sufficient attention is the potential role of stigma. Stigma is a socially constructed process that occurs whereby a person or group possesses an ‘attribute that is deeply discrediting’ ([23], p. 3) that results in discrimination and social devaluation. Stigmatization involves identifying an individual or group as being different, labelling them with unfavorable stereotypes, and delineating the differences between those subjected to stigma and others in society [24]. Stigmatized identities can be ascribed due to physical differences or through association with past events judged negatively by society [25].

In line with this, there is some evidence that acne sufferers perceive greater stigmatization related to their skin than people without acne [26] and that this is associated with impaired psychological functioning [15]. These findings, although rare, are reinforced by findings showing the experience of stigmatization for individuals with other skin conditions, such as leprosy [27], vitiligo [28] and atopic dermatitis [29]. Moreover, there is research evidence demonstrating that others do stigmatize and discriminate against acne sufferers. For instance, a recent study demonstrated that non-sufferers perceive those with acne as unattractive, and anticipate experiencing shame upon developing the condition themselves [30]. Additionally, based on a qualitative study, Magin et al. [6] noted that acne sufferers reported being subjected to bullying, teasing and social exclusion. As such, we know that those with acne are stigmatized, but there is not yet much evidence of specifically how this relates to well-being.

To date, one study demonstrated that higher levels of perceived stigma predicted greater impairment of health-related quality of life, and that perceived stigma contributed more to predicting health-related quality of life than factors typically examined such as gender, age and acne severity [15]. This analysis was the first to examine the effects of perceived stigma on the health-related quality of life of acne sufferers, and the results have not yet been verified in subsequent research. While Liasides and Apergi [15] solely focused on the association between perceived stigma and health-related quality of life, the current study seeks to investigate a wider range of well-being outcomes. In doing so, we seek to contribute meaningfully to literatures on the health outcomes associated with acne, but also stigmatized groups more generally.

Specifically, our study attempts to build on previous research by examining the link between perceived stigma and health-related quality of life, as well as two further well-being outcomes that are of interest in the broader stigmatized identities literature; psychological distress and somatic symptoms. Indeed, an extensive body of research demonstrates that the experience, anticipation and/or internalization of stigma is associated with increased psychological distress [24, 31–34] and physical health problems, including increased somatic symptoms in those who are unemployed (e.g., sleeplessness, headaches, colds; [35, 36]) and a variety of negative physical health outcomes in individuals with HIV/AIDS (e.g., coughing, nausea, chest pain [33]; reduced physical well-being [37]; low CD4 count [38]).

Importantly, while recent developments in this literature often relate to concealable stigmatized identities such as HIV/AIDS, there is also a considerable body of research relating to visible stigma. For example, previous research has illustrated that individuals with visible differences such as race [39], obesity [40] and hair loss [41] are stigmatized and marginalized by society due to their distinctions, and that this stigma is associated with impaired well-being [32]. However, in this body of work there is less focus on skin conditions such as acne, and so the impact of stigmatization on outcomes such as psychological distress and somatic symptoms has not yet been investigated in acne sufferers. The current study will address this gap.

Similarly, in the literature on dermatological conditions, there has so far been less attention on how stigma relates to health-related quality of life in people who suffer from acne, compared to other skin conditions. We currently know more about how stigmatization has been linked with poorer health-related quality of life in studies analyzing skin conditions such as psoriasis [25], vitiligo [28] and atopic dermatitis [29]. As such, it is clear that the current study can meaningfully develop both literatures by examining the capacity for stigma to predict well-being outcomes in acne sufferers.

It should be noted that studies in these two literatures tend to use slightly different well-being outcomes. While some of the psychological literature on stigmatized identities relates to general well-being outcomes such as quality of life [42–43], more often the focus is on psychological distress, specifically referring to depression and anxiety, and to some extent physical health problems including somatic symptoms. Such constructs share similarity with health-related quality of life, which is more often investigated in relation to dermatological conditions [25, 28–29], and which focuses on the impact of the condition on psychological, physical and social domains of well-being. While health-related quality of life is in some senses a broader measure, it also relates explicitly to the dermatological condition, in this case acne. Conversely, studies of stigmatized identities tend to take an overall measure of psychological distress and/or physical health symptoms, and examine to what extent these are predicted by stigmatization. As such, in seeking to build upon and extend both literatures, we argue it is important to investigate how stigmatization of acne contributes to our understanding of all three well-being outcomes: health-related quality of life, psychological distress and somatic symptoms. In the present study, we examine to what extent experienced stigmatization of acne can be used to predict health-related quality of life, psychological distress and somatic symptoms, over and above established predictors of well-being in this group (gender, acne severity, acne location, and medication).

Individuals labelled with stigmatized identities experience shame and dejection due to perceptions that they are not meeting the expectations of wider society [8]. We focus on perceived stigma; that is, people’s perceptions that they have been subject to discrimination due to having acne (similar to experienced stigma). We chose this focus because acne is a visible distinction; as such, other popular measures such as anticipated stigma are less relevant. For example, anticipated stigma is more relevant in studies of concealable stigmatized identities, where many people hide the stigmatizing condition yet still anticipate being stigmatized if their secret was known (e.g., [31]). In relation to the outcomes in our study, we measure health-related quality of life in terms of self-report of how acne impacts participants’ quality of life, as in prior studies on skin conditions [25, 28–29]. We operationalize psychological distress as a composite of the participants’ anxiety and depression levels, as acne sufferers have been documented to exhibit increased anxiety and depression in comparison to non-sufferers [4–5, 21]. In line with previous research [44–45], physical health is operationalized by assessing self-reported somatic symptoms. We predict that stigmatization will contribute significantly towards the prediction of health-related quality of life, psychological distress, and somatic symptoms, over and above other established predictors—specifically gender, acne severity, acne location, and medication.

## Materials and methods

### Procedure

Ethical approval was granted by the Faculty of Education and Health Sciences Research Ethics Committee at the University of Limerick (approval number: 2016_11_05_EHS), and the research was conducted in accordance with the ethical principles of the Declaration of Helsinki. As approved by our Research Ethics Committee, the participants indicated their informed consent by clicking “Continue” to move from the information page to the start of the survey, and by completing the survey. The participants in this cross-sectional study were recruited through convenience sampling. Recruitment emails were sent to all students and staff members on our university’s directory. The emails contained a hyperlink to the survey which participants completed online using Questback software. The participants were required to be at least 18 years of age and have experienced acne at any point two months prior to starting the survey; there were no other inclusion/exclusion criteria. All participants were notified that they were free to withdraw at any stage. They were debriefed following their participation. Participants did not receive compensation for taking part in the study.

### Measures

Each participant initially responded to demographic items and disclosed their gender, age and position (staff or student). Additionally, they were presented with non-mandatory questions regarding the nature of their acne. Specifically, the participants were asked if they consider their acne to be mild, moderate or severe. Similar self-rated measures of severity have been used in previous research investigating the psychological consequences of acne [20, 46]. The participants were also requested to disclose the location of their acne; facial, truncal or facial/truncal. The relation between acne location and psychological distress has been examined in this manner previously [9]. Given that acne patients that are administered treatment have been shown to experience improved psychological functioning [21], participants in the present study were asked if they were taking medication or not.

Next, participants responded to our variables of interest, in the order presented below. In our analyses, gender, acne location, acne severity and use of medication were considered control variables. Perceived stigma served as the predictor variable, while health-related quality of life, psychological distress and somatic symptoms were the outcome variables.

#### Perceived stigma

Perceived stigma was measured using an adapted version of the Day-to-Day Discrimination Scale [39]. The original scale describes nine different types of discrimination and participants indicate how often they have experienced each of these in the past (e.g., ‘People act as if you are inferior.’) In the current study, the instructions requested the participants to indicate on a scale ranging from 1 (*Not at all*) to 5 (*Very often*) how often they feel they have experienced each type of discrimination in the past two months as a direct result of having acne. Additionally, as in a previous study targeted towards a predominantly university-age sample [33], we included six additional items that capture scenarios whereby such individuals commonly experience social devaluation. An example is ‘People not wanting to get involved in an intimate relationship with you’. Total scores could range from 15 to 75, with higher scores representing greater perceived stigma. As in previous research investigating stigmatized identities (e.g., [35]), very high internal reliability was found in the current study (Cronbach’s α = 0.93).

#### Health-related quality of life

The participants’ health-related quality of life was measured using a modified version of the Dermatology Life Quality Index (DLQI; [47]). This 10-item measure consists of questions examining how a person’s skin has affected their life in six domains: symptoms and feelings, daily activities, leisure, school/work, personal relationships and treatments. In the current study, the items and instructions of the questionnaire were modified to examine how the participants’ acne (as opposed to their skin disease in general) has affected their lives in the past two months. The modified version also differed in that one item which includes mention of gardening was altered to refer to attending college or work, as much of our recruitment targeted students and gardening was assumed to be unlikely to be present in a student’s weekly routine. Participants indicated on a scale ranging from 0 (*Not at all*) to 3 (*Very much*) how much each aspect of their lives were affected by their acne. The total scores can range from 0 to 30, with higher scores signifying greater impairment of health-related quality of life. The original DLQI measure has been used regularly in clinical settings [13] and has been previously demonstrated to be an effective measure of the health-related quality of life of acne patients [4, 16]. The modified measure demonstrated high internal reliability in the present study (Cronbach’s α = 0.86).

#### Psychological distress

Psychological distress was assessed by measuring the participants’ levels of anxiety and depression using the Hospital Anxiety and Depression Scale (HADS; [48]). The 14-item scale consists of statements about symptoms of anxiety and depression and participants in the present study indicated the degree to which they had experienced each symptom in the past two months on four-point scales ranging from 0 to 3. Six items were reverse scored, while the anchors varied depending on the item. Total scores can range from 0 to 42 with higher scores indicating higher psychological distress. HADS has been previously used in research examining patients with acne [21, 49]. The measure has been demonstrated to be a valid measure of anxiety and depression symptoms [50]. In the current study, high internal reliability was observed (Cronbach’s α = 0.88).

#### Somatic symptoms

The participants’ somatic symptoms were assessed using the 14-item Physical Health Questionnaire (PHQ; [51]). The participants indicated on a scale ranging from 1 (*Not at all*) to 7 (*All of the time*) how often they experienced each symptom in the past two months. The somatic symptoms included sleep disturbances, headaches, respiratory infections and gastrointestinal problems. As in previous research [35–36, 52], the items were summed into total scores which can range from 14 to 98. One item was reverse scored, with higher total scores indicating poorer somatic health. The measure has been utilized in previous research examining the relationship between perceived facial attractiveness and physical health [45] and has been shown to be a reliable and valid measure of physical health [44, 51]. In the present study, high internal reliability was observed (Cronbach’s α = 0.87).

### Participants

A total sample of 276 acne sufferers at a university in Ireland participated in this study (265 students, 11 staff members). Five participants were excluded after screening for outliers, incomplete responses, and inattentive responding during preliminary analyses, resulting in a final sample of 271 participants. Of this final sample, 81 participants were male (29.9%) and 190 were female (70.1%). Their ages ranged from 18 to 51 years (*M* = 21.63, *SD* = 4.88). Most participants rated their acne as mild (35.4%) or moderate (52.0%), with a smaller proportion considering their acne severe (12.6%). The majority were affected by single-domain facial acne (52.8%) or both facial and truncal acne (39.8%), with single-domain truncal acne affecting the fewest number (7.4%). In terms of treatment, 39.1% of participants were taking medication to treat their acne.

### Statistical analyses

We utilized IBM SPSS 23 to analyze the data. To test our prediction that stigmatization would contribute significantly towards the prediction of health-related quality of life, psychological distress, and somatic symptoms, over and above other established predictors, we conducted three separate hierarchical linear regression analyses. In all of these analyses, gender, acne severity, acne location and medication were entered at Step 1, while perceived stigma was entered at Step 2. Hierarchical regression allows for the comparison of Step 1 and Step 2 models, so it is possible to determine if the change in *R*^2^ between models is significant. In addition, the effect size of any change can be calculated. In the current study, we use Cohen’s *f*^2^ as an indicator of effect size. Cohen advised that 0.02 represents a small effect size, 0.15 a medium effect size, and 0.35 a large effect size [53].

In terms of how control variables were treated, given that both acne severity and acne location had more than two categories, we generated dummy-coded variables to represent these contrasts. Mild acne was utilized as the reference group for acne severity as it was the lowest level of severity measured in the current study. Facial acne was selected as the reference group for acne location as most participants in this study were affected by single-domain acne manifested on the face [54].

## Results

### Preliminary analyses

A total of 472 people clicked the link to the online survey, of whom 276 completed the entire survey (58.5% completion rate). As noted earlier, after initial data screening five participants were excluded from further analyses. The assumptions of multivariate normality, linearity and homoscedasticity were then tested and satisfied through analysis of the histograms, normal P-P plots and scatterplots of the residuals. Although the scores for perceived stigma were positively skewed, transformation was not carried out due to large sample size and as all assumptions were satisfied [55]. We evaluated multicollinearity by assessing the tolerance statistic and variance inflation factor. In accordance with O’Brien’s criteria [56], tolerance values exceeding .10 and variance inflation factor values less than 5 were deemed acceptable—no issues with multicollinearity were identified in the sample.

### Descriptive statistics

The means, standard deviations, and intercorrelations between the measures are presented in Table 1. Mean levels of perceived stigma were relatively low. The mean levels of health-related quality of life impairment appear relatively high, with acne having a very large effect on the participants’ lives [57], although the fact we used a modified version of the DLQI means direct comparisons should not be made. The mean levels of psychological distress would be considered normal to mild [50]. Although there are no classifications available for the PHQ, the mean level was slightly under the midpoint of the scale and can therefore be viewed as moderate.

**Table 1:** Intercorrelates and descriptive statistics.

	**1**	**2**	**3**	**4**	**5**	**6**	**7**	**8**	**9**	**10**	**11**
**1. Perceived stigma**	-										
**2. Health-related quality of life**	.61***	-									
**3. Psychological distress**	.55***	.51***	-								
**4. Somatic symptoms**	.30***	.34***	.58***	-							
**5. Severity: Mild vs. moderate**	.05	.17**	.06	.03	-						
**6. Severity: Mild vs. severe**	.27***	.32***	.22***	.08	.39***	-					
**7. Location: Facial vs. truncal**	-.06	.09	-.01	.03	-.04	.11	-				
**8. Location: Facial vs. both**	.04	.00	.02	.01	.06	-.10	.23***	-			
**9. Medication**	.05	.22***	.06	-.08	.03	.20**	-.11	.01	-		
**10. Gender**	-.07	.13*	.12	.24***	.08	-.12	-.16*	-.08	.06	-	
**11. Age**	-.03	-.02	.09	.03	-.07	.04	-.01	-.12*	-.05	.14*	-
***N***	271	271	271	271	271	271	271	271	271	271	271
***M***	24.52	11.32	13.70	44.76	-	-	-	-	-	-	21.63
***SD***	9.62	6.06	7.40	14.45	-	-	-	-	-	-	4.88
**Min**	15	0	0	14	-	-	-	-	-	-	-
**Max**	75	30	42	98	-	-	-	-	-	-	-

Min = minimum possible value; Max = maximum possible value.

*p < .05.

**p < .01.

***p < .001.

As can be seen in Table 1, perceived stigma was significantly and positively associated with all three well-being outcomes: health-related quality of life, psychological distress and somatic symptoms. Therefore, acne sufferers that reported higher perceived stigma also reported greater health-related quality of life impairment, higher psychological distress levels and more somatic symptoms. In relation to the control variables, gender was found to be significantly correlated with both health-related quality of life and somatic symptoms, with females experiencing greater life quality impairment and more symptoms than males. Acne severity was significantly correlated with health-related quality of life and psychological distress. Acne location was not significantly correlated with health-related quality of life, psychological distress or somatic symptoms, indicating that the site of the participants’ acne is not associated with well-being. Medication was significantly and positively correlated with health-related quality of life, with individuals taking medication experiencing greater impairment than those who were not.

### Hierarchical regression analyses

As outlined in more detail above, three hierarchical multiple regression analyses were conducted to examine our hypothesis that perceived stigma would add to the prediction of health-related quality of life, psychological distress and somatic symptoms of acne sufferers after statistically controlling for gender, acne severity, acne location and medication. All assumptions of hierarchical multiple regression were tested and satisfied during preliminary analyses.

#### Perceived stigma predicting health-related quality of life

As hypothesized, the inclusion of perceived stigma in the hierarchical regression model resulted in a significant increase in explained variance of health-related quality of life after accounting for all other variables (see Table 2). The overall regression model at Step 1 was statistically significant, *F*(6, 264) = 14.69, *p* < .001, accounting for 25.0% of variance in health-related quality of life. Gender, acne severity, acne location and medication were all found to be significant predictors of health-related quality of life.

**Table 2:** Hierarchical regression analyses for variables predicting health-related quality of life, psychological distress, and somatic symptoms.

	Criterion Variables											
Predictor	Health-Related QoL				Psychological Distress				Somatic Symptoms			
	β	*p*	*R*^*2*^	*ΔR*^*2*^	β	*p*	*R*^*2*^	*ΔR*^*2*^	β	*p*	*R*^*2*^	*ΔR*^*2*^
Step 1			.25	.25			.10	.10			.09	.09
Gender	.17	.002			.15	.013			.27	< .001		
Severity: Mild vs. moderate	.32	< .001			.17	.010			.08	.235		
Severity: Mild vs. severe	.43	< .001			.32	< .001			.17	.012		
Location: Facial vs. truncal	.12	.041			-.01	.891			.06	.317		
Location: Facial vs. both	.07	.221			.05	.417			.06	.308		
Medication	.13	.025			-.02	.724			-.13	.040		
Step 2			.52	.27			.34	.24			.18	.09
Gender	.20	< .001			.18	.001			.29	< .001		
Severity: Mild vs. moderate	.21	< .001			.06	.263			.01	.824		
Severity: Mild vs. severe	.23	< .001			.13	.035			.05	.429		
Location: Facial vs. truncal	.17	< .001			.04	.453			.09	.122		
Location: Facial vs. both	.05	.301			.03	.582			.05	.392		
Medication	.14	.002			-.01	.930			-.12	.047		
Perceived stigma	.55	< .001			.52	< .001			.32	< .001		

The overall regression model at Step 2 was statistically significant, *F*(7, 263) = 39.98, *p* < .001, explaining 51.6% of variance in health-related quality of life. The control variables that were significant predictors of health-related quality of life at Step 1 remained significant at Step 2. The introduction of perceived stigma explained an additional 26.5% of variance in health-related quality of life and this change in *R*^2^ was statistically significant, *F*(1, 263) = 143.95, *p <* .001. The effect size for the addition of perceived stigma to the model was large (Cohen’s *f*^2^ = .56). Perceived stigma was significantly and positively associated with higher health-related quality of life, such that higher perceived stigma predicted higher levels of health-related quality of life impairment; standardized *β* = .548, *t*(263) = 12.00, *p* < .001. Further analyses demonstrated that perceived stigma made the largest unique contribution, explaining 26.5% of the variance of health-related quality of life. By comparison, acne severity accounted for 5.2%, gender accounted for 3.8%, acne location accounted for 2.5% and medication accounted for 1.9%. In sum, and as predicted, perceived stigma added to the prediction of health-related quality of life of acne sufferers after statistically controlling for gender, acne severity, acne location and medication. We conducted post-hoc power analysis using G*Power, which showed the power to detect the observed effects at the .001 level was 1.00.

#### Perceived stigma predicting psychological distress

In line with our predictions, the inclusion of perceived stigma led to a significant increase in explained variance of psychological distress after accounting for all other variables (see Table 2). The overall regression model at Step 1 was statistically significant, *F*(6, 264) = 4.78, *p* < .001, accounting for 9.8% of variance in psychological distress. Gender and acne severity were found to be significant predictors of psychological distress. Acne location and medication were not statistically significant predictors of psychological distress.

The overall regression model at Step 2 was statistically significant, *F*(7, 263) = 19.20, *p* < .001, explaining 33.8% of variance in psychological distress. Gender and acne severity remained significant at Step 2, in particular the mild vs. severe comparison. The introduction of perceived stigma explained an additional 24.0% of variance in psychological distress and this change in *R*^2^ was statistically significant, *F*(1, 263) = 95.50, *p <* .001. The effect size for the addition of perceived stigma to the model was large (Cohen’s *f*^2^ = .36). Perceived stigma was significantly and positively associated with higher psychological distress, such that higher perceived stigma predicted higher levels of psychological distress; standardized *β* = .522, *t*(263) = 9.77, *p* < .001. Further analyses demonstrated that perceived stigma made the largest unique contribution, explaining 24.0% of the variance of psychological distress. By comparison, gender accounted for 3.0%, acne severity accounted for 1.1%, acne location accounted for 0.2%; while medication did not uniquely explain additional variance in psychological distress. Therefore, as hypothesized, perceived stigma added to the prediction of psychological distress of acne sufferers after statistically controlling for gender, acne severity, acne location and medication. Post-hoc power analysis using G*Power showed the power to detect the observed effects at the .001 level was 1.00.

#### Perceived stigma predicting somatic symptoms

As predicted, the inclusion of perceived stigma in the hierarchical regression model resulted in a significant increase in explained variance of somatic symptoms after accounting for all other variables (see Table 2). The overall regression model at Step 1 was statistically significant, *F*(6, 264) = 4.56, *p* < .001, accounting for 9.4% of variance in somatic symptoms. Gender, acne severity and medication were found to be significant predictors of somatic symptoms. Acne location was not a statistically significant predictor of somatic symptoms.

The overall regression model at Step 2 was statistically significant, *F*(7, 263) = 8.44, *p* < .001, explaining 18.3% of variance in somatic symptoms. Gender and medication remained significant at Step 2, though acne severity was rendered non-significant when perceived stigma was also taken into account. The introduction of perceived stigma explained an additional 8.9% of variance in somatic symptoms and this change in *R*^2^ was statistically significant, *F*(1, 263) = 28.79, *p <* .001. The effect size for the addition of perceived stigma to the model was small to medium (Cohen’s *f*^2^ = .11). Perceived stigma was significantly and positively associated with higher somatic symptoms, such that higher perceived stigma predicted a greater number of somatic symptoms; standardized *β* = .318, *t*(263) = 5.37, *p* < .001. Further analyses demonstrated that perceived stigma made the largest unique contribution, explaining 8.9% of the variance of somatic symptoms. By comparison, gender accounted for 7.9%, medication accounted for 1.2%, acne location accounted for 0.8% and acne severity accounted for 0.2%. As such, and as predicted, perceived stigma added to the prediction of somatic symptoms of acne sufferers after statistically controlling for gender, acne severity, acne location and medication. Using G*Power, we conducted post-hoc power analysis and found that the power to detect the observed effects at the .001 level was .98.

## Discussion

Previous research shows acne to be stigmatizing and to be associated with impaired quality of life and other well-being outcomes. However, despite the established link between stigma and impaired well-being in other samples, it has not previously been conclusively shown that stigmatization of acne helps to predict impaired well-being, over and above established demographic predictors. The present study addresses this gap by investigating whether acne sufferers’ perceptions of stigmatization significantly predict psychological and physical health outcomes; specifically health-related quality of life, psychological distress, and somatic symptoms. Acne sufferers at an Irish university completed online measures investigating perceived stigma, health-related quality of life, psychological distress and somatic symptoms. As hypothesized, higher levels of perceived stigma predicted greater impaired health-related quality of life, psychological distress and somatic symptoms, over and above gender, acne severity, acne location and medication. As such, the current study reinforces previous research investigating the factors affecting the health-related quality of life of acne sufferers, and presents evidence regarding the relevance of perceived stigma for the psychological distress and somatic symptoms experienced by this group.

### Key findings

Our finding that perceived stigma significantly contributed to predicting impaired health-related quality of life in acne sufferers, over and above the influence of all other variables, illustrates the importance of examining factors other than those typically associated with health-related quality of life impairment in this group. Although the existing psychological and dermatological literature investigating the health-related quality of life of acne sufferers is highly informative, these results indicate that perceived stigma is also a highly important factor worthy of greater consideration in future research. The current study reinforces findings from the study by Liasides and Apergi [15], whereby perceived stigma was the largest contributor to the health-related quality of life of acne sufferers, with factors such as gender and acne location having secondary importance. The current study also provides novel evidence regarding the respective contributions of gender, acne severity, acne location and use of medication to predicting health-related quality of life. Furthermore, our findings add to the relatively sparse literature investigating the association between stigmatization and the health-related quality of life of individuals with visible skin conditions [25, 28–29].

Moving beyond health-related quality of life, the present study is the first to examine the relation between stigmatization and anxiety/depression and physical health problems experienced by acne sufferers. Indeed, the study by Liasides and Apergi [15] did not include anxiety/depression or physical health as outcome measures, meaning our study both supports and advances on their important findings relating to health-related quality of life. By focusing on psychological distress and somatic symptoms, our findings therefore contribute meaningfully to the literature on stigmatized identities more generally. In this literature, outcomes such as psychological distress (and to a lesser extent, somatic symptoms) are quite common, but there has not yet been a focus on groups such as acne sufferers. As such, the finding that greater perceived stigma predicts higher levels of both psychological distress and somatic symptoms extends previous research showing that acne sufferers experience anxiety and depression [49, 58] by accounting for the impact of stigma. The finding is also in line with previous research demonstrating that stigmatized identities are linked with increased physical health problems [33, 40, 59].

Importantly, the findings also provide further support for the comparatively limited amount of studies investigating physical health problems experienced by acne sufferers. Participants in the present study were found to experience similar levels of somatic symptoms to other stigmatized groups (e.g., [35]), which reinforces recent research showing that acne sufferers are likely to develop somatic symptoms other than those directly related to the skin condition itself [7, 60], particularly if they feel stigmatized.

It is also interesting to note the contribution of the current study to the understanding of established predictors of impaired well-being outcomes in acne sufferers. In our study, as in previous research [16–17], female participants were found to experience greater impairment than males. It has previously been suggested that females encounter increased social pressure to possess clear skin, and are consequently more concerned about the appearance of their acne than males [61]. The study also reinforces previous work (e.g., [1], [18]) demonstrating that increased acne severity contributes to decreased health-related quality of life, with individuals that self-rated their acne as severe experiencing the highest impairment. Acne sufferers’ perceptions of their acne severity are an indicator of self-confidence and body image satisfaction [20], which in turn could have hindered health-related quality of life. Interestingly and in contrast to previous research [22], individuals with single-domain truncal acne experienced greater health-related quality of life impairment than those with facial acne, suggesting that decreased visibility did not result in decreased impairment. We speculate that this may be because although truncal acne is largely concealable, it has nevertheless been linked with greater self-consciousness of sexual and bodily appearance [61]. Finally, the results indicate that participants taking medication experienced higher health-related quality of life impairment than those who were not, possibly due to increased awareness of their condition.

### Limitations and future directions

The present study addresses clear gaps in the literature on health-related quality of life in sufferers of acne and other skin conditions, and in the stigmatized identities literature. However, there are naturally several limitations that should be considered in future research. As this study was cross-sectional in nature, it is not possible to make inferences about causality. In order to discern causality, future research could investigate the effects of stigmatization and factors influencing the health-related quality of life, psychological distress and somatic symptoms of acne sufferers on a longitudinal basis. It is also worth noting that the participants in this study were university students recruited through convenience sampling. Previous research has indicated that recruiting participants in this manner can lead to biased results, as university students often have less-crystallized attitudes than individuals in later adulthood, as well as greater cognitive abilities and stronger tendencies to comply with authority [62]. Similarly, the relatively low completion rates could have led to non-response bias [63], while systematic drop-out patterns can be influenced by survey design features and particular personality traits [64].

Moreover, self-reported measures were employed to determine well-being and the nature of acne. Self-reported measures can be influenced by factors such as social desirability and the participants’ levels of stress and mood during completion [65]. As such, the results should be evaluated with caution, as the participants self-diagnosed their acne and the extent of their condition. Given that acne is a complex disease and manifests in various forms, the participants’ evaluations of their acne may have diverged from their actual clinical diagnosis. Future research could diagnose acne severity more precisely through assessment via a global measure such as the Leeds-Revised Acne Grading Scale [66]. It would also be useful to have participants report specifically what type of medication they are using, if any, to control for this in a more precise manner.

Although perceived stigma was observed to substantially predict the health outcomes measured in the current study, the mean levels of perceived stigma were relatively low. This may not be considered surprising given the high pervasiveness of the condition and mild nature of the symptoms in comparison to other facial deformities perceived negatively by society, such as post-burn scarring and severe facial trauma [67]. Indeed, similar levels of stigma have been identified in previous research investigating the effects of stigma on psychological and physical health (e.g., [33]).

The levels of perceived stigma could also be explained by participants’ ages, as previous research has indicated that acne sufferers are subjected to less teasing and bullying as they enter adulthood [6]. As such, we might see higher levels of perceived stigma in a sample of teenage acne sufferers. Therefore, it is advisable that future research investigates this issue using a more diverse age distribution. In addition, one study has suggested that individuals without a previous history of acne are more likely to experience health-related quality of life impairment due to their newly experienced symptoms [15]. Future research could investigate the manner in which a sufferer’s history of acne and the duration of acne affects psychological and physical health.

Further, while most participants self-rated their acne as mild or moderate, it should be noted that acne severity was positively associated with higher levels of perceived stigma. As such, in order to fully establish the degree to which stigmatization affects the psychological and physical health of acne sufferers, future research could focus specifically on the impact of perceived stigma on the health outcomes of sufferers with clinically severe acne as diagnosed by medical professionals. It would also be possible to extend the results of the current study by recruiting a sample with more varied experience in terms of acne location, as very few participants reported having single-domain truncal acne.

Finally, it was interesting that females were found to experience increased health-related quality of life impairment and more somatic symptoms than males, and indeed this was consistent with previous research. However, over two-thirds of the acne sufferers in our study were female. This is perhaps unsurprising, given that females have been documented to be afflicted more commonly by acne than males after adolescence [68]. However, ideally future studies should investigate the effects of acne stigma with a sample including a greater number of males, to establish more conclusively whether gender differences are apparent.

## Conclusions and implications

The findings of the present study align with previous research showing that individuals with visible distinctions viewed negatively by society are subjected to stigma, which is associated with impaired psychological and physical well-being. In terms of theoretical implications, our analyses further elucidate the important role of stigma in predicting the well-being of individuals with chronic skin conditions. Research on the health-related quality of life of acne sufferers has tended to focus on demographical factors, and research on links between stigmatization and well-being has largely focused on disadvantaged groups with more conspicuous visible distinctions such as race [39] and obesity [40]. It is therefore significant for both these literatures that our study indicates stigmatization contributes considerably to our understanding of the well-being of acne sufferers, in terms of health-related quality of life, psychological distress and somatic symptoms. To date, few studies have examined the association between stigma and the psychological and physical health of this group, and thus the empirical evidence presented in the current study is both valuable and relevant.

The research also has considerable practical relevance and a number of notable clinical implications. Our findings suggest that interventions that attempt to counter the effects of stigma could improve the psychological and physical health of acne sufferers. For example, introducing classes at primary level that teach strategies on how to cope with stigmatization could prepare young people for the inevitable increase in the scrutiny of their physical appearance as they enter adolescence. Similarly, workshops could be developed to help adult acne sufferers cope with perceived stigmatization, which could in turn decrease health-related quality of life impairment, psychological distress and somatic symptoms. Additionally, medical professionals and counsellors alike should place increased emphasis on social factors such as stigma when selecting suitable methods for managing the consequences of the condition.

Although skin conditions are commonly overlooked as mere cosmetic problems, the symptoms experienced by sufferers are often unpredictable, difficult to manage, and can have a considerable impact on self-esteem, body image and overall well-being. The current study substantiates the large body of research demonstrating that acne sufferers are susceptible to an array of health consequences due to their condition. Our findings illustrate the importance of examining the potential influence of social factors such as stigma that have not received sufficient attention in previous years. By developing a more complete understanding of the social factors affecting acne sufferers, it could be possible to improve the techniques for managing the diverse range of emotional and health consequences associated with the condition.
